# Three‐dimensional mapping of junctional beats during atrioventricular nodal reentrant tachycardia cryoablation

**DOI:** 10.1002/joa3.12768

**Published:** 2022-08-11

**Authors:** Ermenegildo De Ruvo, Vincenzo Coscia, Mattia Petrungaro, Leonardo Calò

**Affiliations:** ^1^ Policlinico Casilino Rome Italy; ^2^ Cardiology Department University of Campania L. Vanvitelli Caserta Italy; ^3^ Cardiology Department Sant'Andrea Hospital, University of Rome Sapienza Rome Italy

**Keywords:** atrioventricular nodal reentrant tachycardia, catheter ablation, cryoablation, junctional beats, three‐dimensional mapping

## Abstract

Cryoablation of slow pathway doesn't usually cause junctional beats. If this occurs, the nearness to AV compact node is supposed. 3d electroanatomical mapping during this unusual finding may help to clarify the relationship between junctional beats (JBs) during cryomapping/cryoablation and Koch's triangle.

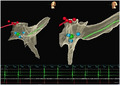

Cryoablation of slow pathway does not usually cause junctional beats. If this occurs, the nearness to AV compact node is supposed. Three‐dimensional electroanatomical mapping during this unusual finding may help to clarify the relationship between junctional beats (JBs) during cryomapping/cryoablation and Koch's triangle.

Histological aspects of the two techniques could explain this difference. As a matter of the fact, cryoenergy produces a homogeneous, sharper lesion. Otherwise, RF lesion is less well circumscribed, with serrated edges.^1^

In literature, there is only one case reporting junctional beat during cryoablation.^2^ The authors assumed that the closeness to AV compact node caused junctional beats. In our case, we have witnessed JBs during cryoablation in the setting of a three‐dimensional mapping, thus we can exactly localize the point where JBs were elicited.

A 60‐year‐old woman was hospitalized for recurrent episodes of palpitations. She was treated for AVNRT with slow pathway RF ablation 14 years ago. The slow pathway was ablated in the posteroseptal region obtaining numerous junctional beats. After RF application AVNRT was no more inducible. During the follow‐up, 24 h ECG monitoring revealed a narrow regular QRS tachycardia with a cycle length of 490 ms and also a nocturnal complete AVB with junctional escape rhythm at rate of 55 bpm. Patient was enrolled after informed consent was obtained, according to the procedure established by the Ethics Committee of our institution.

Preliminary electrophysiological study has showed normal basal parameters: (AH 71 ms, HV 43 ms). The Wenckebach cycle length was 530 ms. A typical slow–fast AVNRT was inducible with programmed atrial stimulation. Because of the risk of iatrogenic AV block, we have chosen to treat the recurrence with cryoablation. No alarming response has been registered during cryomapping (at −40°C) in every site of ablation point.

We started ablation from the anterior border of the CS ostium^3^ because we feared AV block. During second application slow pathway cryoablation we elicited JBs, which represent a typical response and a target of RF ablation but are unusual during cryoablation.

Helped by NavX three‐dimensional electroanatomical mapping, we can show the exact location of this unusual finding (Figure 1 and Video S1). Of course, cryoablation was stopped when junctional beats occurred and a new posterior site was found, successfully completing the slow pathway ablation. Surprisingly, junctional beats are posteriorly located. Our atypical response to cryoablation confirms the complex nature of compact AV node.

**FIGURE 1:**
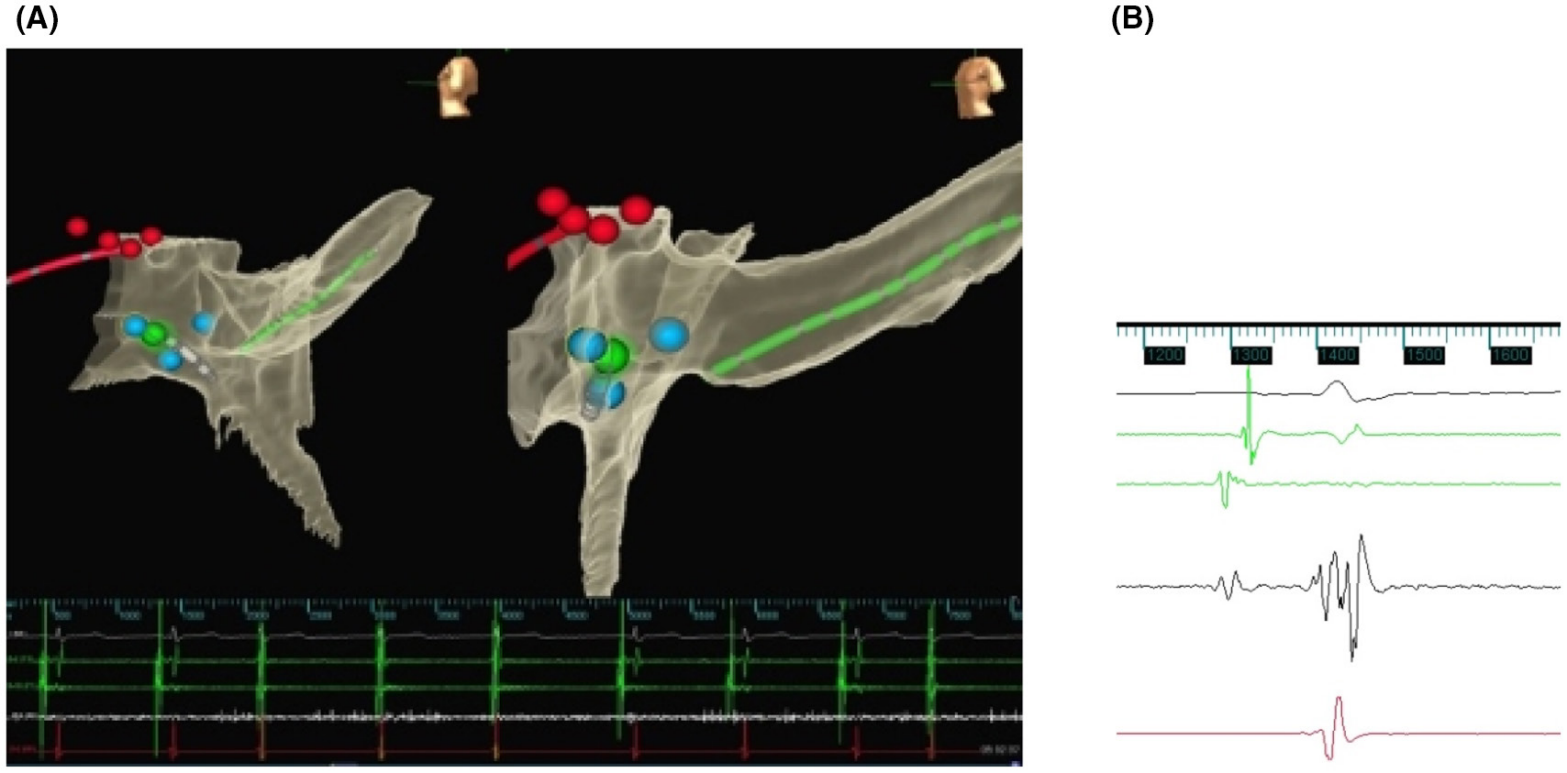
(A) Three‐dimensional mapping of junctional beats during cryoablation. (B) Local EGM before cryoablation.

This is the first case in which we can exactly localize the site of JBs during cryoablation. We stopped cryoablation supposing iatrogenic AV node damage, but we cannot exclude that junctional beats can be a target even in cryoablation of AVNRT. Further studies and three‐dimensional mapping are needed to understand clinical impact of junctional beats during cryoablation.

Figure 1A shows a LAO and LL NavX three‐dimensional maps. His bundle is tagged in red. Cryoablation points are tagged in blue. The green point shows the ablation site where JBs were elicited.

Figure 1B shows local EGM before cryoablation, with a 2:1 V:A ratio. In this typical ablation site, we find JBs during cryoablation (video attached).

## FUNDING INFORMATION

The authors have not declared a specific grant for this research from any funding agency in the public, commercial or not‐for‐profit sectors.

## CONFLICT OF INTEREST

The authors declare that they have no known competing financial interests or personal relationships that could have appeared to influence the work reported in this paper.

## Supporting information


Video S1
